# Ultrafast capillary electrophoresis isolation of DNA aptamer for the PCR amplification-based small analyte sensing

**DOI:** 10.3389/fchem.2015.00049

**Published:** 2015-08-12

**Authors:** Emmanuelle Fiore, Eric Dausse, Hervé Dubouchaud, Eric Peyrin, Corinne Ravelet

**Affiliations:** ^1^Département de Pharmacochimie Moléculaire UMR 5063, Centre National de la Recherche Scientifique, University Grenoble AlpesGrenoble, France; ^2^Laboratoire ARNA, Institut National de la Santé et de la Recherche Médicale U869, Université BordeauxBordeaux, France; ^3^Laboratoire de Bioénergétique Fondamentale et Appliquée, Institut National de la Santé et de la Recherche Médicale U1055, University Grenoble AlpesGrenoble, France

**Keywords:** split aptamer, real time PCR, amplification, capillary electrophoresis, small molecule

## Abstract

Here, we report a new homogeneous DNA amplification-based aptamer assay for small analyte sensing. The aptamer of adenosine chosen as the model analyte was split into two fragments able to assemble in the presence of target. Primers were introduced at extremities of one fragment in order to generate the amplifiable DNA component. The amount of amplifiable fragment was quantifiable by Real-Time Polymerase Chain Reaction (RT-PCR) amplification and directly reliable on adenosine concentration. This approach combines the very high separation efficiency and the homogeneous format (without immobilization) of capillary electrophoresis (CE) and the sensitivity of real time PCR amplification. An ultrafast isolation of target-bound split aptamer (60 s) was developed by designing a CE input/ouput scheme. Such method was successfully applied to the determination of adenosine with a LOD of 1 μM.

## Introduction

A new class of recognition components, aptamers, were discovered by Ellington (Ellington and Szostak, 1990) and the combinatorial approach of systematic evolution of ligand by exponential enrichment (SELEX) was developed for separation of these oligonucleotides (Irvine et al., 1991). Nucleic acid aptamers have become during the last decade highly important molecular recognition tools for bioanalysis purpose as exemplified by the very abundant literature data. To date, one of the most important challenges to meet is related to the improvement of the aptamer-based assay specificity and sensitivity.

To improve the sensitivity and specificity of aptasensors, sandwich-based assays with aptamers have been developed and employed for sensitive and selective detection of proteins. Because sandwich assays require the simultaneous binding of two capture elements (Heyduk and Heyduk, 2005) they are greatly specific, providing them adequate for use immediately in complex media, such as blood serum or plasma. In order to detect a small molecule which usually only binds to one aptamer, one strategy is splitting the aptamer into two fragments to establish the sandwich interaction. This elegant approach has been extensively used in the small target sensing area using different transduction methods including colorimetric, fluorescence, and electrochemical techniques (Zuo et al., 2009; Lin et al., 2011; Du et al., 2012; Zhu et al., 2012, 2014; Rogers et al., 2015). However, the major problem of the split aptamer approach is related to the significant decrease in the ternary complex stability upon splitting of the functional oligonucleotide, (Sharma and Heemstra, 2011; Sharma et al., 2012; Kent et al., 2013) leading to low sensitivity assays.

The amplified detection issue appears especially crucial for the small analyte sensing as the dissociation constant for the small target-aptamer complexes is typically in the (sub)micromolar range so that the sensitivity potentialities are intrinsically limited. Reported amplified aptasensing strategies are based on the DNA amplification through conventional molecular biology tools like polymerase chain reaction (PCR) or rolling circle amplification (RCA) techniques (Liu et al., 2015). The DNA amplification approach appears to be particularly popular due to the specific nature of nucleic acid aptamers which appears especially suitable for the facile incorporation of the amplifiable template into their molecular recognition sequence. Moreover, this feature constitutes a major advantage relatively to the antibodies as the immune-PCR and related methods necessitate notably the difficult-to-control synthesis of the antibody-DNA chimera (Adler et al., 2005). Numerous PCR or RCA amplified aptamer-based assays have been recently reported for the detection of proteins or cells/bacteria (Yoshida et al., 2012; Wu et al., 2015) but only very few examples have been described for the small analyte determination (Huang et al., 2013; Yang et al., 2015b).

Similarly to immuno-PCR assays, nucleic acid amplification-based aptasensing methods require generally a capture step of an oligonucleotide (aptamer in sandwich assays or complementary strand in structure-switching assays) and the subsequent isolation stage of the amplifiable DNA template. This process involves in most cases magnetic beads or microplate wells. However, the heterogeneous format of such reported methods is associated with significant limitations such as the requirement of surface DNA immobilization, the occurrence of non-specific interaction between surfaces and DNA (leading to high background signal) and the involvement of time-consuming washing cycles.

A very interesting alternative strategy, initially reported by Le and co-workers for protein detection, is based on the combination of the capillary electrophoresis (CE) and PCR amplification methods (Zhang et al., 2006). Compared to the surface-based separation methods, CE possesses very attractive advantages such as homogeneous format, very high separation efficiency (from typically 100,000 to 1 million theoretical plates), very low volume sample requirements (in the nanoliter range), fully automated instrumentation and multiplexed ability. Unfortunately, the described approach cannot be adapted to the small analyte sensing due to the difficulty to separate the amplifiable target-bound aptamer fraction from the free aptamer. Indeed, the electrophoretic mobility of the two species in zone CE is very close because of the same mass/charge ratio. In addition, as a consequence of its weak stability, the small molecule-aptamer complex is expected to dissociate during its residence time in the capillary under typical CE analysis format.

**Figure 1:**
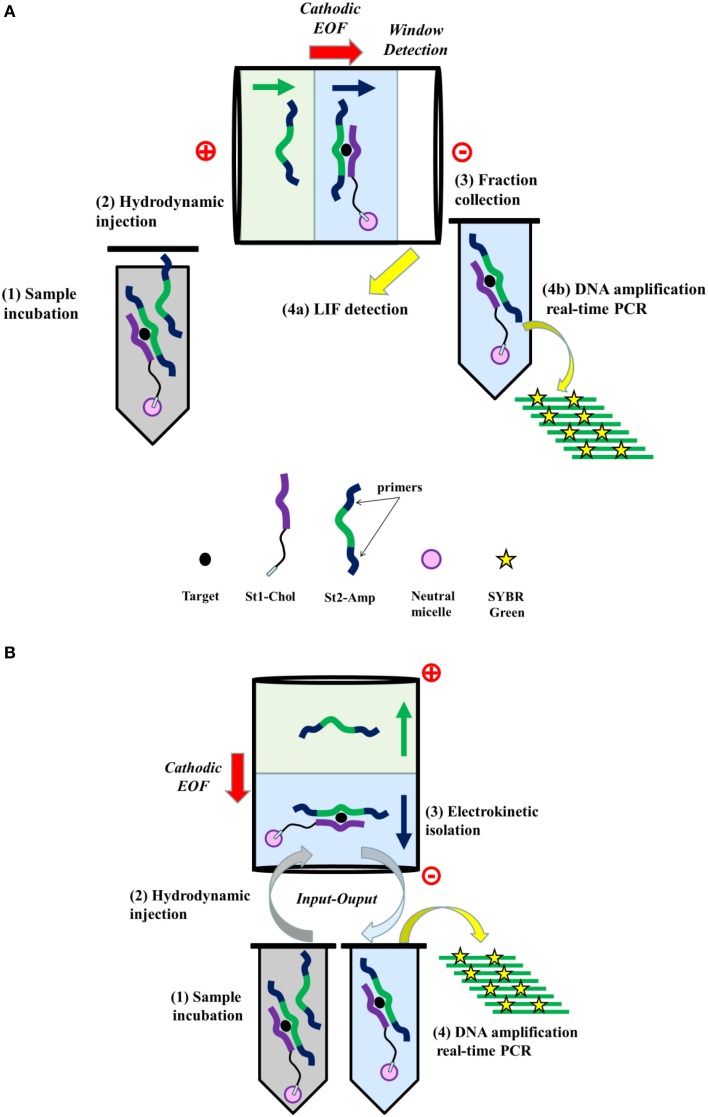
**General principle of the aptamer assay based on the isolation of target-bound DNA template by capillary electrophoresis and its subsequent amplification by real time PCR**. **(A)** Conventional CE separation format. **(B)** Input/output CE separation format.

Herein, we describe a new homogeneous sandwich-type aptamer assay dedicated to the DNA amplification-based sensitive detection of small analyte target that combines CE advantages for the isolation of the target-bound DNA template and its subsequent amplified quantitation by real-time PCR (RT-PCR). The general principle is illustrated in Figure 1.

First, the aptamer is cut in two DNA strands (St_1_and St_2_) to work under the sandwich-like mode, i.e., the two split aptamer fragments are able to assembly in presence of target (Stojanovic et al., 2000). Second, primers are introduced at extremities of the St_2_ fragment (St_2_-Amp) in order to generate the amplifiable DNA component. Third, the St_1_ strand is cholesteryl-tagged (St_1_-Chol). As previously demonstrated, (Zhu et al., 2010), a shift of the electrophoretic mobility of the cholesteryl-modified DNA species can be reached by micellar electrokinetic chromatography (MEKC) through the well-known drag-tag concept (Won et al., 2005).

In a first stage, the proof of concept was demonstrated on a conventional separation format (system A, Figure 1A) with laser induced fluorescence (LIF) detection to verify the complex formation (Figure 1A) and was further compared to amplified quantitation by RT-PCR. Subsequently, the target-dependent DNA template isolation was achieved using a capillary input/output format (system B, Figure 1B) with a new improved sandwich design in order to (i) accelerate analysis, (ii) limit the complex dissociation phenomena, (iii) reduce interference of the unbound St_2_-Amp species, and (iv) enhance the efficiency of fraction collection.

## Experimental

### Reagents

β-D-adenosine (A), tris (hydroxymethyl) aminomethane (Tris), lauryl ether (Brij 35) were acquired from Sigma-Aldrich (Saint-Quentin, France). Sodium chloride (NaCl) and Magnesium chloride hexahydrate (MgCl_2_·6H_2_O) were purchased from Chimie-Plus laboratories (Bruyères de Pouilly, France) and Panreac Quimica (Barcelona, Spain), respectively. HCl and NaOH were provided by Carlo Erba (Val de Reuil, France). All chemicals were at least of analytical grade. Water was purified using a Purite Still Plus system (Thame, UK) fitted with a reverse osmosis cartridge. The DNA oligonucleotides (Table 1) were made by Eurogentec (Belgium), only St_2_-Amp (A and B), F- St_2_-Amp (A and B) were synthesized by Eurofins Genomics (Germany).

**Table 1:** **Sequences used in the study**.

**Name**	**Sequences (5′–3′)**
**SYSTEM A**	
**St**_1_**-CholA**	Chol-ACCTGGGGGAGTA
**St**_2_**-AmpA**	CCAACCGTGAAAAGATGATGGTGCGGAGGAAGGT**CTTAGGTCTATCCCGATGCC**
**F-St**_2_**-AmpA**	F-CCAACCGTGAAAAGATGATGGTGCGGAGGAAGGT**CTTAGGTCTATCCCGATGCC**
**For primA**	CCAACCGTGAAAAGATGATGG
**Rev primA**	**GGCATCGGGATAGACCTAAG**
**F-St**_1_**A**	F-ACCTGGGGGAGTA
**St**_2_	TGCGGAGGAAGGT
**SYSTEM B**	
**St**_1_**-CholB**	Chol-ATACCTGGGGGAGTATATAAT
**St**_2_**-AmpB**	CACACAGGAAACAGCGTATGACAATTATAGCGGAGGAAGGTAT**CCGTAGCCCTATCTGGATTGC**
**F-St**_2_**-AmpB**	F-CACACAGGAAACAGCGTATGACAATTATAGCGGAGGAAGGTAT**CCGTAGCCCTATCTGGATTGC**
**For primB**	CACACAGGAAACAGCGTATGACA
**Rev primB**	**GCAATCCAGATAGGGCTACGG**
**F-St**_1_**-CholB**	Chol-ATACCTGGGGGAGTATATAAT-F

Abbreviations: Chol, cholesteryl tag; F, fluorescein label. For, Forward, Rev, Reverse, Prim, Primer. Complementary sandwich bases are shown in red. Complementary bases between template and reverse primer are shown in bold type.

### Sample preparation

D-adenosine and oligonucleotides stock solutions were made in pure water and froze at −20°C. Stock solutions of oligonucleotides were diluted every day in a concentrated incubation buffer. For the titration curve, the target concentration varied from 0, 1, 5, 10, 50 to 2000 μM in the incubation buffer. The sample solutions were denaturated at 80°C during 5 min, kept at room temperature for 15 min, and left at 4°C for 15 min to reach equilibrium of the sample prior to the CE injection and separation.

The incubation buffer is composed of a buffered solution 10 mM Tris-HCl (pH 7.5), 50 mM NaCl, and 10 mM MgCl_2_ with 0.5 mM of Brij 35 for system A and 20 mM Tris-HCl (pH 7.5), 25 mM NaCl, and 5 mM MgCl_2_ containing 0.5 mM of Brij 35 for system B. The samples were composed of F-St_2_-Amp(A or B), Rev prim(A or B), and St_1_-Chol(A or B) oligonucleotides at a final concentration of 1, 1, and 40 μM, respectively for system **A** and 20, 20, and 800 nM, respectively, for system B.

### Capillary electrophoresis separation

A P/ACE MDQ instrument from Beckman Coulter (Fullerton, CA) utilizing LIF detection with excitation at 488 nm and emission at 520 nm was used. Fused silica capillary, 75 or 50 μm inner diameter, 365 μm outer diameter, was purchased from Polymicro Technologies (Phoenix, AZ). Fused silica capillaries were prepared before analysis by achieving different washes at 20 psi: 1 M NaOH for 5 min, water for 5 min, and background electrolyte (BGE) for 30 min. The washing process between runs was performed at 20 psi with 1 M NaOH (2 min), water (2 min), and BGE (5 min).

#### System A

The BGE was composed of 20 mM Tris, 25 mM NaCl, 0.5 mM Brij pH 7.8. A 50 μm inner diameter (*i.d.*) capillary (total and effective lengths of 30 and 20 cm, respectively) was employed. The sample (either the equilibrium mixture of split aptamers with Rev prim and adenosine or split aptamers only with Rev prim) was injected in the inlet side of the capillary by a pressure at 0.7 psi for 16 s. The capillary temperature was left at 20°C and migration was achieved at 15 kV. Seven fractions were collected all the minutes at the outlet of the capillary into PCR vials containing 30 μL BGE.

#### System B

The BGE was composed of and 20 mM Tris, 25 mM NaCl, 5 mM MgCl_2_, 0.5 mM Brij pH 7.5. A 75 μm inner diameter (*i.d.*) capillary (total and effective lengths of 50 and 40 cm, respectively) was used. The sample prepared as above was injected in the inlet side of the capillary by a pressure at 0.7 psi for 4 s. A buffer solution was injected into the capillary by a pressure at 0.3 psi for 2 s. The capillary temperature was kept at 20°C and migration was carried out at 12 kV. Only one fraction of 1 min was collected into a PCR vial.

### RT-PCR

After collection, fractions were analyzed through real-time PCR (RT-PCR) using the Applied Biosystem StepOne™ RT PCR system. RT-PCR was done with two primers: the forward and the reverse primer (see Table 1 for each system). The primers were drawn using Primer express 3.0 software (Applied Biosystem) to limit complementarity to each other, in order to decrease non-specific amplification of self-dimerizing primers. The Mfold web server was also used to limit non-specific interaction between St_1_-Chol and St_2_-Amp and with primers.

For amplification, 20 μL of PCR mix was prepared with SYBR® Green PCR core reagents consisting of 2 μL of 10X SYBR Green PCR buffer, 2.4 μL of 25 mM MgCl_2_ solution, 1.6 μl of dNTP mix, 0.1 μL of AmpliTaq Gold DNA polymerase (1 U/μL), 0.2 μL of Amperase (1 μL/μL), 2 μL of 10 μM of each primer (forward and reverse primer), 2 μL of collected fraction as St_2_-Amp template, and 7.7 μL H_2_O. Real-time PCR was carried out in MicroAmp™ fast reaction tubes, closed with strip caps (Applied Biosystems). The thermal cycling regime was: initial denaturation for 10 min at 95°C, and then cycling for 15 s at 95°C, 60 s at 60°C for system A or 5 s at 60°C, 32 s at 72°C for system B repeated 50 times. In order to follow the St_2_-Amp amplification, the fluorescence variations of a double-stranded DNA binding dye, SYBR Green I, were observed by Applied Biosystem StepOne™ RT-PCR machine (Applied Biosystems). All measurements were realized in triplicate, and the threshold cycle (Ct) values were determined from the mean of each Ct-value.

### Fluorescence polarization

The binding buffer for fluorescence anisotropy assay (FA) consisted of 10 mM Tris buffer pH 7.5, 50 mM NaCl, 10 mM MgCl_2_ for system A and 20 mM Tris buffer pH 7.5, 25 mM NaCl, 5 mM MgCl_2_ for system B. The acid nucleic reserve solutions were prepared in pure water at 10^−5^M and stored at −20°C. The sample solutions of F-St_1_(A or B), St_2_(A or B), St_2_-Amp(A or B), and Rev primer (A or B) were prepared from the reserve solutions by dilution with concentrated binding buffer (2.5 X) for FA. The sample solutions were heated at 80°C for 5 min and then kept at 25°C for 30 min in order to attain stability prior to use. The target solutions were made in water. For the titration curve, the target concentration changed from 15 to 2000 μM. Before use, all sample solutions were sieved through 0.2 μm membrane filters.

To create the dose-response curves, individual wells were prepared by mixing the solution of St_2_,St_2_-Amp, or St_2_-Amp-Rev primer duplex containing different amounts of target with the F-St_1_solution at 25°C (final volume = 100 μL), resulting in oligonucleotide final concentrations of 400 nM except for the probe fixed at 10 nM. Blank wells were prepared by addition of 100 μL of the binding buffer in the microplate. The microplate was disposed immediately into the microplate device for the reading of anisotropy.

The fluorescence anisotropy (*r*) was determined by the microplate software, as classically reported:

$$\begin{array}{l} r=\frac{I_{vv}-GI_{vh}}{I_{vv}+2GI_{vh}} \end{array}$$

where *I*_*vv*_ and *I*_*vh*_ are the vertically and horizontally polarized elements of the emission after excitation by vertically polarized light. The correction factor *G* was calculated by the instrument from standard solutions conforming to the supplier's instructions.

Fluorescence anisotropy variation Δ*r* was determined as comes after:

$$\begin{array}{l} \Delta r=r-r_{0} \end{array}$$

Where *r* is the anisotropy of the mixture (St_2_,St_2_-Amp, or St_2_-Amp-Rev primer duplex with F-St_1_) and *r*_0_ is the anisotropy in absence of analyte. Δ*r*_*max*_ is the maximal fluorescence anisotropy difference, obtained with adenosine.

$$\begin{array}{l} relative\Delta r=\frac{\Delta r\times 100}{\Delta r_{\max}} \end{array}$$

## Result and discussion

### Target-induced split aptamer formation and RT-PCR amplification

To establish the proof of principle, we chose the anti-adenosine aptamer (Apt-A). This option was inspired by the well-known binding and structural properties of Apt-A in previous works (Lin and Patel, 1997). It has been previously shown that the parent 25-mer Apt-A splitted into two single stranded DNA pieces (St_1_ and St_2_) is able to recognize the adenosine target (Zhu et al., 2012).

The design of the template (St_2_-Amp) was first assessed. It was of crucial importance to evaluate the impact of the incorporation of the amplifiable sequences at the St_2_ extremities on the formation of the ternary complex. In this context, the binding ability of St_2_-Amp was compared with that of unmodified St_2_ by fluorescence anisotropy using fluorescent St_1_ strand as probe. The addition of the second modified piece (St_2_-Amp) at different concentrations (80, 200, 400, 600 nM) did not change the fluorescence anisotropy. This is due to the absence of affinity between the two DNA fragments without adenosine. When adenosine was incorporated into the reaction medium (2000 μM), the formation of the ternary complex (F-St_1_A/Ade/St_2_-AmpA) caused an augmentation in the FA signal, dependent on the variation in the molecular size between the free F-St_1_A fragment and the ternary complex. The best result was obtained for 1:40 stoichiometry (with 10 nM F-St_1_A) (Supplementary Figure S1). However, as compared with the initial sandwich (F-St_1_/St_2_), a weaker assay response (data not shown) and a different profile curve were observed with the new sandwich dedicated to RT-PCR (Supplementary Figure S2). The presence of primers at the St_2_ extremities increased significantly the apparent dissociation constant *K*_*d*_ of the ternary complex (>500 vs. ~90 μM), probably originating from steric hindrance effects and/or non-specific hybridization. To overcome this limitation, the 3′ end-primer sequence was blocked with its complementary strand (Rev primA) (1:1 stoichiometry), allowing the restoration of the original binding properties (apparent *K*_*d*_ ~ 90 μM, Supplementary Figure S2).

**Figure 2:**
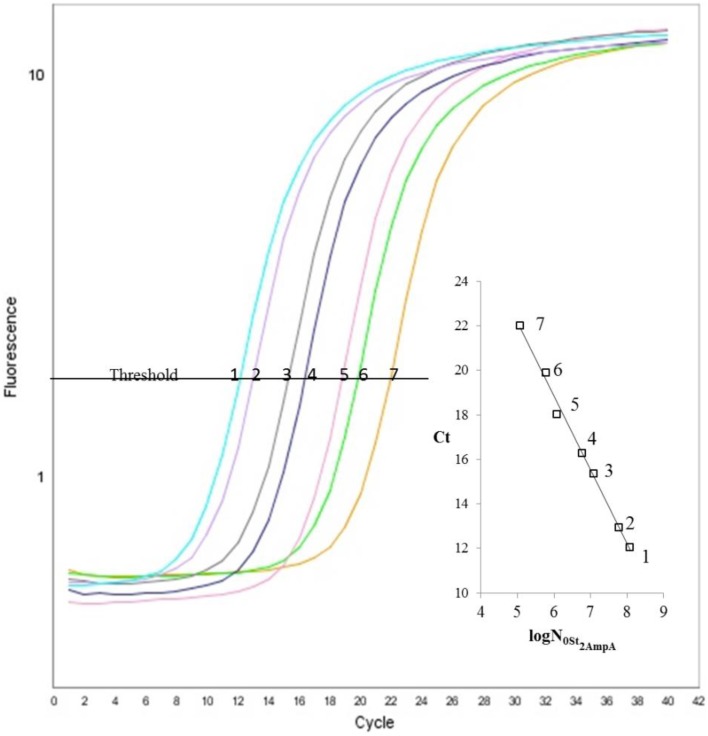
**Amplification curves obtained by real-time PCR for St_2_-AmpA using SYBR Green I**. Fluorescent signal represents the emission of the fluorescent signal from SYBR Green, divided by the emission of the reference dye (ROX) at the given time. Serial dilution of St_2_-AmpA of 1.2 × 10^8^ to 1.2 × 10^5^ number of species/20 μL Legend: 1.2 × 10^8^ (1, turquoise), 6.02 × 10^7^ (2, purple), 1.2 × 10^7^ (3, gray), 6.02 × 10^6^ (4, blue), 1.2 × 10^6^ (5, pink), 6.02 × 10^5^ (6, green), 1.2 × 10^5^ (7, yellow). Inset: Efficiency plot of St_2_-AmpA serial dilution series. Displays the Ct-values vs. log initial number of St_2_-AmpA (N_0_), with a linear regression fit of *R*^2^ = 0.994. The slope of the line is −3.32 providing an efficiency of 100.8 ± 0.44% using *E* = 10^(−1∕slope)^−1.

The adequate choice of both the annealing temperature and the primer concentration is judicious to underground the primer artifacts during the RT-PCR technique. For St_2_-AmpA, the best primer concentration was 1 μM (data not shown). The quantitative SYBR Green I fluorescence signal amplification data are shown in Figure 2. In addition, the threshold cycle values and the logarithm of the amount of St_2_-AmpA are drawn in inset (Figure 2). In this practicability work, a correlation between the Ct**-**value and the quantity of the St_2_-AmpA was discerned. The correlation coefficient was determined (*R* = 0.994). The linear standard fitting was detected from 120,000 molecules to 120 million molecules. The slope value was determinate as −3.32, which was very similar to the value of 100% PCR amplification efficiency. However, it is of interest to note that the addition of St_1_-Chol A in the RT-PCR mixture decrease the amplification efficiency (Table 2). It is well known that the structure of the oligonucleotide target (e.g., stem and loop secondary DNA/RNA structure) has a considerable consequence on the efficiency of the PCR (Bustin et al., 2009). The presence of the complementary strand of the amplicon likely inhibited slightly the Taq polymerase activity.

**Table 2:** **Results of RT-PCR calibration**.

**Systems**	**Slope**	***R*^2^**	**Efficiency (%)**
St_2_-AmpA	−3.32	0.994	100.8
St_2_-AmpA + St_1_-CholA^a^	−4.27	0.986	71.4
St_2_-AmpA + St_1_-CholA + adenosine^b^	−4.36	0.986	69.5
St_2_-AmpB + St_1_-CholB + Rev primB^c^	−3.51	0.989	92.5

Serial dilution of different systems with 1.2 × 10^7^ to 1.2 × 10^4^ number (except for St_2_-AmpA alone, see legend Figure 2) of St_2_-AmpA or B species/20 μL.

a With [St_2_-AmpA]/[St_1_-CholA] ratio = 1/40.

b With [St_2_-AmpA]/[St_1_-CholA] ratio = 1/40 and 2000 μM of adenosine.

c With [St_2_-AmpB]/[Rev primB]/[St_1_-CholB] ratio = 1/1/40 and 5 μM of adenosine.

### Adenosine assay by conventional CE separation format (system A)

**Figure 3:**
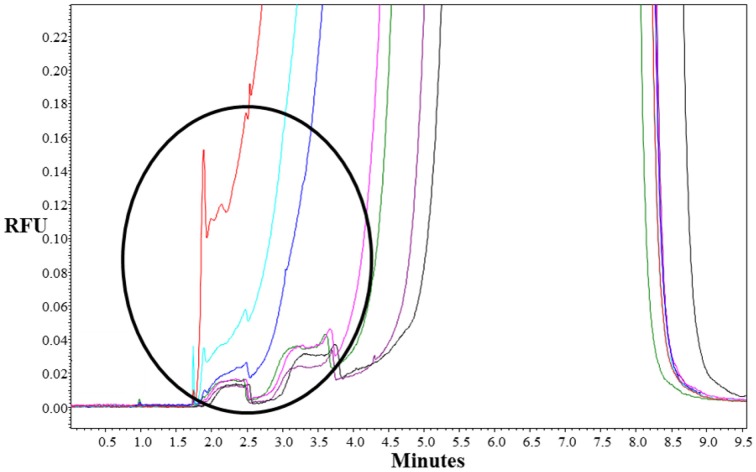
**Representative electropherograms of system A**. Preincubated samples: (purple) F-St_2_-AmpA and Rev primA (1 μM of each)**;** F-St_2_-AmpA, Rev primA, and St_1_-CholA (1, 1, and 40 μM, respectively) with 0 μM (black), 50 μM (green), 100 μM (pink), 500 μM (blue), 1000 μM (turquoise), and 2000 μM (red) of adenosine. Incubation buffer: 10 mM Tris-HCl (pH 7.5), 50 mM NaCl, and 10 mM MgCl_2_ containing 0.5 mM of Brij 35. CE conditions: running buffer, 20 mM Tris-HCl (pH 7.8), 25 mM NaCl, and 0.5 mM Brij 35; capillary, 50 μm *i*.*d*.; effective length, 20 cm; uncoated, fused silica capillary with LIF detection; applied voltage, 15 kV; sample injection, 0.7 psi for 16 s; capillary temperature, 20°C.

To choose the meantime for fraction collection, at first, we analyzed the separation of St_2_-AmpA/Ade/St_1_-CholA complex from the St_2_-AmpA/St_1_-CholA in presence of Rev primA by using a fluorescently F-St_2_-AmpA as a probe. The CE-LIF analyses of mixture with and without adenosine were compared under MEKC conditions. The applied voltage, capillary temperature, and effective length, were respectively 15 kV, 20°C, and 20 cm. A running buffer composed of 20 mM Tris-HCl (pH 7.8), 25 mM NaCl, and 0.5 mM Brij 35 which is a neutral surfactant (critical micelle concentration comprised between 40 and 100 μM) (Tang et al., 2008) was used. A new peak appeared at a lower migration time with the addition of target in the preincubated sample (for 500, 1000, 2000 μM of adenosine, Figure 3). This latter corresponded to the F-St_2_-AmpA/Ade/St_1_-CholA species as the consequence of its rooting into the Brij micelles that migrated faster than F-St_2_-AmpA. The St_1_-CholA and Rev primA species, comigrating with the complex and F-St_2_-AmpA, respectively, were not detected. The complex displayed a low stability because of its dissociation shape for all the adenosine concentration. The fluorescence intensity was measured at around 3 min and normalized by dividing the fluorescence difference obtained with and without target by **Δ**F_max_ (maximal fluorescence difference obtained in presence of target).

For the RT-PCR analysis, we collected fractions all the minutes and each fraction was subjected to amplification. The best fraction allowing the biggest difference between Ct**-**values without and with target was analyzed by using signal normalization.

**Figure 4:**
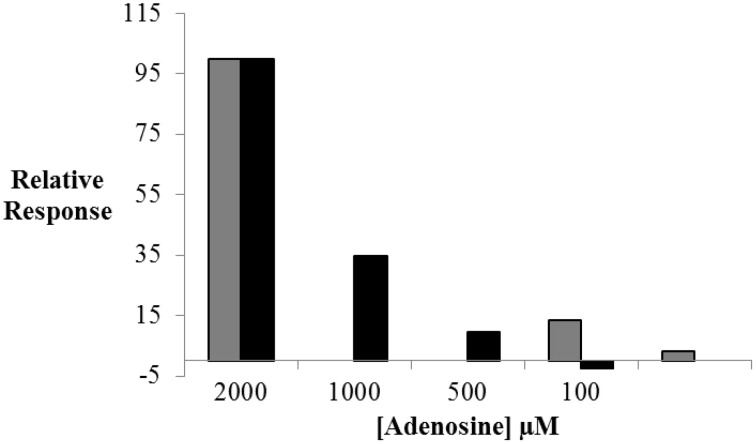
**The relative response in ΔF (black) and Δ*Ct* (gray) values plotted against adenosine concentration**. Relative ΔF = ((ΔF × 100)/ΔF_max_) and ΔF = F - F_0_; ΔF_max_ is the maximal fluorescence difference, obtained in presence of target. Relative ΔCt = ((ΔCt × 100)/ΔCt_max_) and ΔCt = Ct_0_-Ct; ΔCt_max_ is the maximal threshold cycle difference, obtained in presence of target. Operating conditions are as specified in Figure 3.

As shown in Figure 4, the assay sensitivity obtained with RT-PCR analysis was significantly improved (by a factor of ~5) relative to that retrieved with LIF-detection. No target binding to the split aptamer was observed below 500 μM of adenosine using fluorescence measurements whereas 50 μM could be easily detected by amplification of St_2_-AmpA. In order to limit the background signal obtained without target (Ct of about 21) the amount of St_2_-AmpA was decreased to 20 nM. Unfortunately, no significant changes were obtained (data not shown).

### Improved adenosine assay (system B)

Despite the interesting features of this assay, its low sensitivity constituted a major drawback for subsequent sensing applications. In order to improve the sensor performances, we aimed to limit the dissociation complex during the CE analysis.

First, a new design of the split aptamer was constructed by addition of six base pairs at the 5′-end and one base pair at the 3′-end of the St_1_ and St_2_ aptamer (Table 1). Aptamers are generally defined by a well-characterized stem-loop secondary structure. Addition of several base pairs from the terminal stem could show a more stabilized secondary structure of the aptamer which would limit the dissociation complex phenomena (Nozinovic et al., 2014).

Second, a new ultrafast capillary isolation of the template was introduced by using a capillary input/output format (Figure 1B). As previously shown, St_1_-Chol, St_2_-Amp, and target species were incubated in the reaction solution containing neutral micelles. In presence of target, the two split aptamer DNA strands associated to form the St_1_-Chol/target/St_2_-Amp ternary complex. The equilibrium mixture was then hydrodynamically injected at the inlet of a bare fused silica capillary prefilled with the micellar phase-containing buffer (“input”). The magnitude of the cathodic electroosmotic flow (EOF) was adjusted to allow transport of the free and target-bound St_2_-Amp species in opposite direction. For absolute electroosmotic velocity lower than the free oligonucleotide absolute electrophoretic velocity, unbound St_2_-Amp was electrokinetically captured inside the capillary by migrating toward the anodic side. In contrast, due to its cholesteryl group-driven interaction with the cathodic EOF-moving neutral micellar phase, the ternary complex was recovered in a new collection vial at the capillary inlet (“output”). The bound St_2_-Amp collected fraction in 60 s was subsequently submitted to RT-PCR step for the amplified monitoring of the strand-to-complex change dependent on the target concentration in the sample.

Third, Mg^2+^ was added in the BGE and reaction solution as the formation of the target-aptamer complex requires the presence of such divalent cation (Da Costa et al., 2013). However, magnesium cation is also known to reduce the electrosmosis phenomenon by direct interaction with capillary wall silanoate groups. In order to allow the migration of free St_2_-Amp and complex in opposite migration, the magnitude of EOF had was adjusted by controlling both the electrolyte pH and Mg^2+^ concentration by using LIF detection method and 5′-end fluorescein labeled St_2_-Amp oligonucleotide as probe. Under 0.5 mM Brij 35 conditions, the ternary complex moved toward the cathode while the free St_2_-Amp migrated in direction of the anode for a magnesium concentration of 5 mM and an electrolyte pH of 7.5.

**Figure 5:**
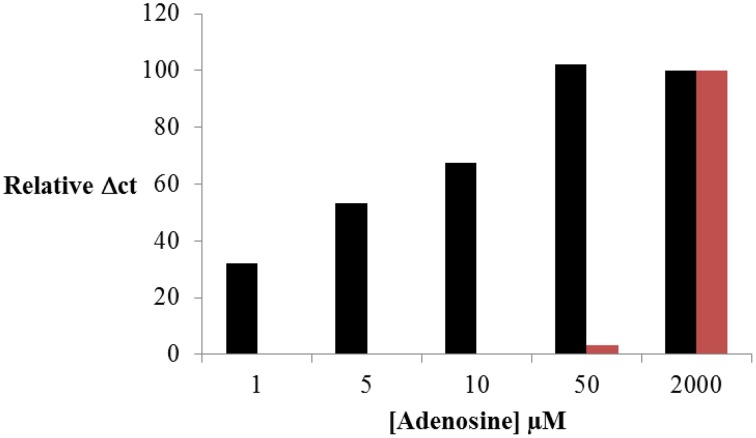
**Adenosine sensing by RT-PCR using ultrafast isolation by CE of St_2_-AmpB/Ade/St_1_-CholB complex (Figure 1B, black) and conventional CE (Figure 1A, red) of St_2_-AmpA/Ade/St_1_-CholA complex**. Operating conditions for CE and RT-PCR are presented in Experimental Section.

The results obtained were presented in Figure 5. As previously shown, the formation of the ternary complex and the optimal conditions were preliminary estimated by fluorescence anisotropy (Supplementary Figure S3). The efficiency of St_2_-AmpB amplification was also checked by RT-PCR (Table 2). Assay response (Relative **Δ***Ct*) increased as the adenosine concentration enhanced from 0 to 50 μM. Below 50 μM, signal saturation was observed. This ultrafast scheme was able to detect 1 μM of adenosine. This is 50-fold lower than the LOD obtained under the conventional separation design (Figure 5). The analytical performance of the present sensing platform appears to be also better than most of the split aptamer approaches (see SI of Yang et al., 2015a). If we compared only LOD obtained for adenosine, the values were estimated at 15 (Zhu et al., 2012), 6 (Bai et al., 2014), and 1 μM (Yang et al., 2015a) for systems employing fluorescence detection. The value obtained in this study is better than or comparable to these aptamer-split methods (except for the LOD of 12 nM determined by Liu and co-authors using self-assembly quantum dot detection), (Liu et al., 2013).

## Conclusion

To the best of our information, this work represents the first presentation of RT-PCR amplification strategy for split aptamer assay dedicated to small analytes. This assay displays some attractive features. Firstly, the assay can be operated in complete homogeneous format by mixing the split aptamer, and others reagents (Brij 35, Taq polymerase, primers), which offers an appropriate method for simple and cost-efficient adenosine detection. The use of RT-PCR with its ability to quantify and gauge minute amount of oligonucleotides in a broad range of samples with its practical simplicity, speed and specificity made it the touchstone for nucleic acid quantification. Secondly, the dissociation of the complex is significantly reduced through the introduction of the input/output scheme, allowing the fast template isolation (<1 min) which is advantageous as compared to the time consuming surface-based separation methods. This CE isolation may be developed for the design of other apta-sensors. Thirdly, given rapid advances in split aptamer engineering technologies, (Kent et al., 2013) it occurs that the split aptamer-RT-PCR platform might serve a new universal platform for selective detection of a broad range of small targets.

## Conflict of interest statement

The authors declare that the research was conducted in the absence of any commercial or financial relationships that could be construed as a potential conflict of interest.
