# Supplementary material for: Ultrafast capillary electrophoresis isolation of DNA aptamer for the PCR amplification-based small analyte sensing
Source: Front Chem. 2015 Aug 12;3:49. doi: 10.3389/fchem.2015.00049 (PMC4533002; doi:10.3389/fchem.2015.00049)

Supporting Information

Ultrafast Capillary Electrophoresis Isolation of DNA Aptamer for the PCR Amplification-Based Small Analyte Sensing

Emmanuelle Fiore,[a] *Eric Dausse,*[b] *Hervé Dubouchaud*[c], *Eric Peyrin*[a*] , *Corinne Ravelet*[a*]

[a]*Univ. Grenoble Alpes, DPM UMR 5063, F-38041 Grenoble*
CNRS, DPM UMR 5063, F-38041 Grenoble, France

[b]*Laboratoire ARNA, Université Bordeaux, Inserm U869, F-33076 Bordeaux, France*

[c]*Univ. Grenoble Alpes, Laboratoire de Bioénergétique Fondamentale et Appliquée, INSERM U1055, F-38041 Grenoble, France*

[*]*Corresponding authors: Email:* eric.peyrin@ujf-grenoble.fr; corinne.ravelet@ujf-grenoble.fr

Figure S1: Fluorescence anisotropy change Δr of F-St$_1$A in presence of 2000 µM adenosine under different St$_2$-AmpA concentrations: 80 nM, 200 nM, 400 nM, 600 nM. F-St$_1$A: 10 nM. Binding buffer conditions: 10 mM Tris-HCl, pH 7.5, 50 mM NaCl, 10 mM MgCl$_2$; reaction temperature 25°C.

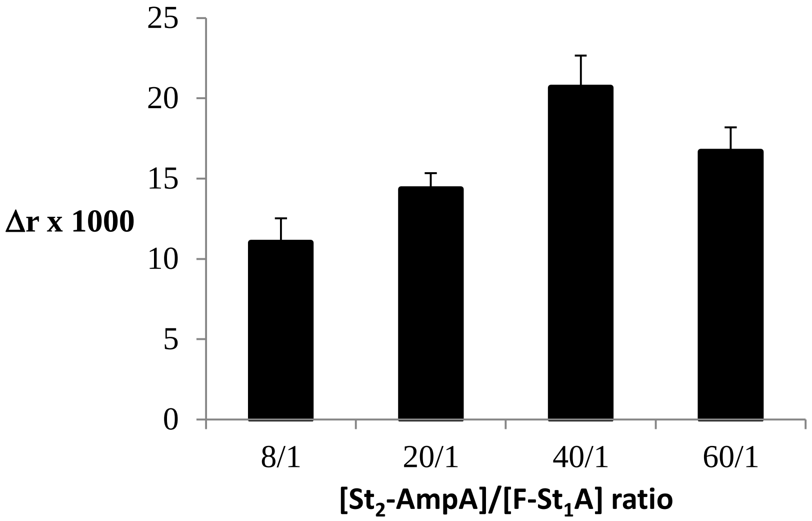

Figure S2 The relative Δr value plotted against the adenosine concentrations using the F-St$_1$A-St$_2$A split aptamer (filled square), F-St$_1$A-St$_2$-AmpA split fragments (open circle) and F-St$_1$A-St$_2$-AmpA-Rev primA hybrid (filled circle). F-St$_1$A: 10 nM, St$_2$AmpA: 400 nM, Rev primA: 400 nM. Binding buffer conditions: 10 mM Tris-HCl, pH 7.5, 50 mM NaCl, 10 mM MgCl$_2$; reaction temperature 25°C.

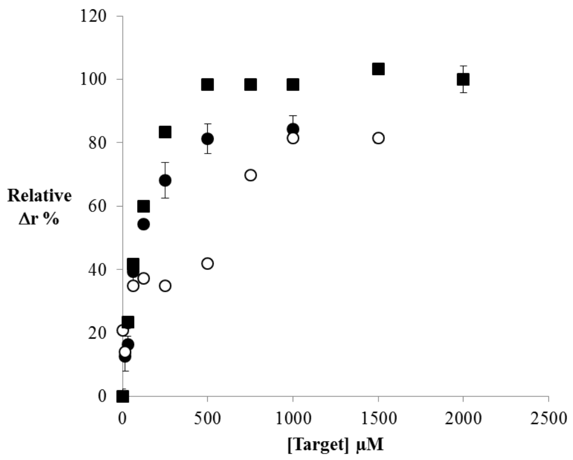

Figure S3: Fluorescence anisotropy change Δr of F-St1-CholB (10 nM) with St_2-AmpB (400 nM) in presence of 125 µM adenosine under different Rev PrimB concentrations: 100 nM, 400 nM, 1600 nM. Binding buffer conditions: 20 mM Tris-HCl, pH 7.5, 25 mM NaCl, 5 mM $MgCl_2$; reaction temperature 25°C.

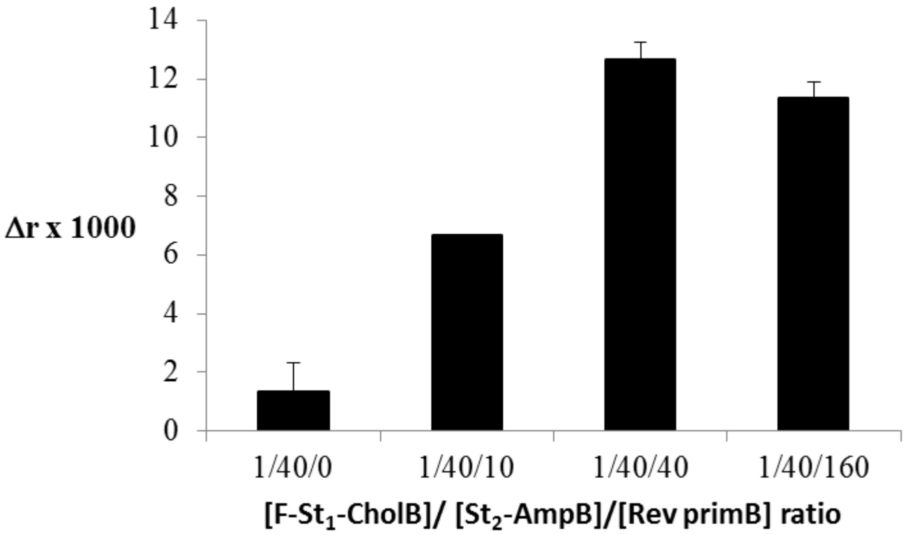

Supplement: Supplementary file 1 [file Presentation1.PDF]
